# Effect of PBAN on Pheromone Production and Biosynthetic Pathway of the Major Sex Pheromone Component of *Proeulia auraria* Clarke (Lepidoptera: Tortricidae)

**DOI:** 10.3390/insects17101037

**Published:** 2026-10-09

**Authors:** Luis Reyes-García, Tomislav Curkovic, Astrid Gajardo, Felipe Gonzalez, Beatriz Valenzuela, Marcos Flores-García

**Affiliations:** 1 Departamento de Ciencias Básicas, Facultad de Ciencias, Universidad Santo Tomás, Viña del Mar 2561780, Chile; 2 Facultad de Ciencias Agronómicas, Universidad de Chile, Santiago 8820808, Chile; tcurkovi@uchile.cl (T.C.); astrid.gajardo@ug.uchile.cl (A.G.); felipe.gonzalez.s@ug.uchile.cl (F.G.); 3 Escuela de Tecnología Médica, Facultad de Salud, Universidad Santo Tomás, Viña del Mar 2561780, Chile; bvalenzuela9@santotomas.cl; 4 Departamento de Horticultura, Facultad de Ciencias Agrarias, Universidad de Talca, Campus Lircay, Talca 3460000, Chile; marcos.flores@utalca.cl

**Keywords:** isotopic tracers, chain shortening, β-oxidation, Δ11 desaturase, fatty acid precursors, GC-MS analysis

## Abstract

*Proeulia auraria* (Lepidoptera: Tortricidae) is a native Chilean moth and a significant pest of several fruit crops. Its sexual communication relies on pheromones, of which (*E*)-11-tetradecenyl acetate (E11-14:OAc) is the major component, but the mechanisms regulating its production and biosynthetic pathways are poorly understood. We studied the effect of pheromone-biosynthesis-activating neuropeptide (PBAN) on pheromone production and its biosynthesis through isotopic labeling using deuterated fatty acids, which were topically applied to the pheromone gland. Gas chromatography–mass spectrometry (GC-MS) analysis was then performed. PBAN application increased E11-14:OAc by up to eight times. In labeling experiments with [16,16,16-^2^H_3_]hexadecanoic acid (D_3_-16:acid), the mass spectrum of E11-14:OAc showed a shifted ion at *m*/*z* 197 and a natural ion at *m*/*z* 194. These results indicated the incorporation of three deuteriums into the terminal methyl group and that chain shortening from palmitic acid to the 14-carbon skeleton occurs through β-oxidation from the carboxyl end. Deuterium incorporation was also detected after D_3_-18:acid and D_3_-14:acid application. The findings provide the first direct evidence regarding the precursors and direction of chain shortening in the pheromone biosynthesis of *P. auraria*. The information contributes to our understanding of its chemical communication and could lay the foundation for the development of pheromone-based control tools.

## Simple Summary

Moths use chemical signals called pheromones to attract mates. *Proeulia auraria* is a moth that is native to Chile and has become a significant pest of fruit crops, such as apples, grapes, and blueberries. Its sex pheromones are a mixture in which (*E*)-11-tetradecenyl acetate is predominant. This study investigated how this pheromone component is regulated and produced. We first injected females with pheromone-biosynthesis-activating neuropeptide (PBAN), which is a natural substance that activates pheromone production. As a result, the amount of pheromone increased up to eight times above normal levels. We next labeled fatty acids with deuterium to allow us to trace their pathway and then directly applied them onto the pheromone-producing gland. This approach revealed that the pheromone is synthesized from common fatty acids such as palmitic acid, which are shortened through β-oxidation. These results expand our understanding of the mechanism that this moth uses to produce its pheromones and how PBAN activates their synthesis by increasing pheromone levels. This foundational knowledge is essential for understanding the chemical communication of this moth species.

## 1. Introduction

Sex pheromones are fundamental chemical signals in the reproductive communication of insects that play a central role in the recognition of potential mates and in reproductive isolation between closely related species [1]. In lepidopterans, these signals typically consist of mixtures of volatile compounds that are derived from fatty acids. Alcohols, aldehydes, and acetates with linear chains of 10 to 18 carbons are predominant compounds and are known as type I pheromones [2,3]. The biosynthesis of these compounds occurs in the female pheromone gland and involves a series of enzymatic transformations. These transformations include desaturation, chain modification, and functional conversion, which determine the identity and proportion of the components in the pheromone blend [4,5].

In many moth species, pheromone-biosynthesis-activating neuropeptide (PBAN) regulates pheromone production and acts on the pheromone gland by stimulating the metabolic pathways that are responsible for synthesizing pheromone components [5,6]. The action of PBAN has been documented in different lepidopteran families, in which its topical application induces or increases the production of sex pheromones, which illustrates its key role in the physiological regulation of these chemical communication systems [7,8]. Studies on biosynthetic pathways have frequently used isotopically labeled precursors to identify metabolic intermediates and elucidate the enzymatic processes involved in the formation of pheromonal compounds [9,10,11].

The moth family Tortricidae includes numerous species with sex pheromones that mainly correspond to unsaturated 12–14–carbon compounds, which often have double bonds at the Δ11 position [4]. In this group, the genus *Proeulia* comprises several species that are native to South America and are associated with fruit crops [12]. *P. auraria* in particular is considered a quarantine pest for chilean exports of fresh apples, grapes, blueberries, and citrus [13]. Studies have identified the main components of its sex pheromones, which are composed of a mixture of unsaturated 14-carbon derivatives, and the major component is (*E*)-11-tetradecenyl acetate (E11-14:OAc) [14,15].

There is still limited information about the physiological mechanisms that regulate pheromone production in *P. auraria* and the biosynthetic pathways that lead to the formation of the components of its pheromone blend. Understanding these processes is relevant for both the study of chemical communication in Lepidoptera and the development of pheromone-based tools for pest management [16,17]. In this context, the objective of the present study was to evaluate the effect of the neuropeptide PBAN on pheromone production in females of *P. auraria*. Furthermore, the biosynthetic pathway was investigated in regard to the main components of its pheromone blend through isotopic labeling experiments and chemical analysis of pheromone gland extracts.

## 2. Materials and Methods

### 2.1. Chemicals

PBAN (*Helicoverpa assulta*/*Heliothis zea*; Cat. No. 4039571.0500) was obtained from Bachem Americas Inc. (Torrance, CA, USA) through Fisher Scientific (Thermo Fisher Scientific, Madrid, Spain). Deuterium-labeled fatty acids, including palmitic acid-16,16,16-D_3_ and stearic acid-18,18,18-D_3_ (98 atom% D), were obtained from Sigma-Aldrich (St. Louis, MO, USA). Tetradecanoic acid-14,14,14-D_3_ (CAS No. 62217-71-4) was purchased from LGC Standards (Teddington, UK). A mixture of 37 fatty acid methyl ester standards (Supelco 37 Component FAME Mix, CRM47885) and a homologous series of *n*-alkanes (used for retention index calculations) were also obtained from Sigma-Aldrich. All solvents used for chemical analyses were of chromatographic grade. Dimethyl sulfoxide (DMSO; ≥99.9%, Sigma-Aldrich, St. Louis, MO, USA) was used as the solvent for the application of labeled fatty acids, and phosphate-buffered saline (PBS) was used for PBAN solution preparation.

### 2.2. Insects

Larvae of *P. auraria* were collected in October 2024 from infested foliage in a vineyard (*Vitis vinifera* L.) in Nancagua (34°40′28.71″ S 71°12′09.86″ W), Libertador General Bernardo O’Higgins Region, Chile. Specimens were transported to the Laboratory of Pest Behavior and Chemical Ecology (CEQUIP) of the Faculty of Agronomical Sciences at the University of Chile (La Pintana, Santiago). The specimens’ identity was confirmed in accordance with Cepeda and Cubillos [18]. Larvae were separated according to instar and placed in groups of three in 30-mL DART plastic cups, which contained 15 g of artificial diet for Lepidoptera (Stonefly Heliothis Diet, Ward’s Science), as reported by Zaviezo et al. [19].

The cups were kept in climatic chambers (Legrandtherm, Santiago, Chile) at 25 ± 0.5 °C and 40–60% relative humidity with a photoperiod of 16:8 (L:D). Pupae were sexed and kept individually in Petri dishes until emergence. Adult identification was confirmed according to González [20]. Adults were placed in mating arenas (500-mL plastic cups covered with aphid-proof mesh) at a male:female ratio of 3:1 and fed with a 5% sugar solution via a cotton wick [21]. The couples mated inside, females laid eggs on the inner cup walls, and then adults were removed.

The first-instar larvae were collected immediately after hatching and reared on the same diet as described until pupation. Pupae were sexed [20] and sent to the laboratory at Universidad Santo Tomás in Viña del Mar. Collection and handling were authorized by the Chilean Agricultural and Livestock Service (SAG; permit No. 1394/2024). *P. auraria* is not a protected species, and all procedures complied with Chilean regulations.

### 2.3. PBAN Application

One- and two-day-old virgin females of *P. auraria* were used for PBAN bioassays since they start calling behavior right after emergence [14]. For all bioassays, three females were used for each sampling time (*n =* 3). Before treatment, the insects were immobilized at −12 °C for 3 min to facilitate handling [22]. PBAN was dissolved in PBS (137 mM NaCl, 2.7 mM KCl, 10 mM phosphate buffer, pH 7.4) to obtain a final concentration of 16.6 pmol µL^−1^. A volume of 0.3 µL of this solution was injected into the abdominal region (between the 5th and 6th sternites) of each insect using a microsyringe at a dose of approximately 5 pmol PBAN per insect. All injections were performed during the photophase (between 09:00 and 14:00 h). Control individuals received the same volume of PBS without PBAN.

After treatment, the insects were maintained under laboratory conditions, and the pheromone glands were excised between 1 and 6 h after PBAN application for chemical analyses. Additional experiments were conducted using mated females with mating status verified by direct observation of copulation. In these assays, the insects received the same PBAN treatment described in the preceding paragraph, but no labeled precursors were applied. The pheromone glands were excised at 1–4 h after injection and extracted using the same procedure described for virgin females.

### 2.4. Application of Labeled Fatty Acids

To investigate the biosynthetic origin of pheromone components, labeled fatty acids were applied to virgin females of *P. auraria*. Before exposure, individuals were immobilized at −12 °C for 3 min to facilitate handling. Solutions of the respective labeled fatty acids were prepared in DMSO at a concentration of 40 µg µL^−1^, and 0.3 µL of each solution were applied directly onto the pheromone gland of individual females using a microsyringe. Control insects received the same volume of DMSO without labeled precursor.

The following deuterium-labeled fatty acids were applied individually: D_3_-14:acid (6 females), D_3_-16:acid (6 females), and D_3_-18:acid (7 females). After precursor application, the females were maintained for 45 min to allow uptake of the labeled fatty acids by the pheromone gland. PBAN was then injected into the abdominal region to stimulate pheromone biosynthesis, as described in Section 2.3. The insects were then maintained for an additional 2 h before gland extraction. The pheromone glands were excised and immersed in hexane for 10 min to extract pheromone components before chemical analysis. The glands were extracted and analyzed individually.

### 2.5. Chemical Analysis

Gas chromatography (GC) analyses were performed using an Expec Technology GC-2000 system (Hangzhou, China) equipped with a flame ionization detector (FID). Analyses were conducted using a fused-silica capillary column (RTX-5, 30 m × 0.25 mm i.d.; Restek, Bellefonte, PA, USA) with nitrogen as the carrier gas. Gas chromatography–mass spectrometry (GC–MS) analyses were carried out using a Shimadzu QP-2010 system (Shimadzu Corporation, Kyoto, Japan). Samples were analyzed using an RTX-5 capillary column (30 m × 0.25 mm i.d.; Restek, Bellefonte, PA, USA), with helium as the carrier gas. Injections were performed in splitless mode with the injector temperature set at 250 °C. The oven temperature program started at 50 °C (held for 5 min) and increased to 270 °C at a rate of 8 °C min^−1^. Mass spectra were acquired in electron impact (EI) mode at 70 eV with a scan range of *m*/*z* 70–500.

Compound identification was based on comparison of mass spectra with the NIST/EPA/NIH Mass Spectral Library [23] and calculated retention indices obtained using a homologous series of *n*-alkanes. Identifications were further confirmed by comparison with authentic reference standards. Reference standards of methyl (*Z*)-11-tetradecenoate, methyl (*E*)-11-tetradecenoate, (*E*)-11-tetradecenoic acid, tetradecyl acetate, (*E*)-11-tetradecenyl acetate, and (*E*)-11-tetradecen-1-ol were synthesized using standard organic synthesis protocols including acetylation, reduction with LiAlH_4_, Jones oxidation, and BF_3_-methanol esterification. All compounds were purified by column chromatography and characterized by GC-MS and nuclear magnetic resonance (^1^H NMR) spectroscopy. Detailed procedures are available in the doctoral thesis of Reyes Garcia [21]. Fatty acid methyl ester standards from the Supelco 37 Component FAME Mix were used as chromatographic references for fatty acid analyses.

### 2.6. Quantification and Data Analysis

Quantification of pheromone components was performed using gas chromatography with flame ionization detection (GC-FID). Peak areas were integrated using chromatographic software associated with the instrument, and compound amounts were calculated based on external calibration curves generated. Synthetic (*E*)-11-tetradecenyl acetate was used as a reference standard. Calibration curves were prepared in the range of 0.01–90.0 ng (R^2^ = 0.9999). For isotopic labeling experiments, the incorporation of labeled precursors into pheromone components was evaluated by examining the corresponding molecular ion signals in mass chromatograms obtained by GC–MS analyses. To prioritize temporal resolution in this time-course experiment, the design used *n =* 3 biological replicates per time point. Accordingly, results are presented descriptively as the mean ± standard deviation (SD) while avoiding inferential statistics, which are not applicable at this sample size. Graphical representations were performed in RStudio using R software (R Core Team, version 4.5.1) and the ggplot2 package [24].

### 2.7. Pheromone Gland Extraction and Derivatization

After the incubation period, insects were killed by freezing, and pheromone glands were excised individually. Each gland was immersed in 30 µL of hexane for 10 min to extract pheromone compounds, after which the solvent extract was transferred to a clean vial for analysis. To analyze the fatty acid precursors remaining in the gland tissue, the extracted gland was immersed in 30 µL of chloroform. The extract was treated with 30 µL of a 20% boron trifluoride–methanol solution and heated at 60 °C for 3 h to convert the fatty acids into their corresponding methyl esters. After cooling to room temperature, 50 µL of water and 50 µL of hexane were added. The organic phase was recovered, dried over anhydrous sodium sulfate (Na_2_SO_4_), and used for GC analyses.

Double-bond positions in unsaturated compounds were determined by chemical derivatization with dimethyl disulfide (DMDS) using a scaled-down version of a reported procedure [25]. Briefly, an aliquot of the methylated extract (~10 µL) was reacted with DMDS (10 µL) and iodine solution in diethyl ether (10 µL, 5% *w*/*v*) as a catalyst, and the mixture was incubated at 50 °C overnight. The reaction was quenched by adding one drop of 10% aqueous sodium thiosulfate solution, followed by the addition of hexane (40 µL). The organic phase was separated, concentrated to approximately 5–10 µL, and analyzed by GC–MS using an RTX-5 column under the conditions described above. Each pheromone gland was processed individually, and all extracts were analyzed separately.

## 3. Results

### 3.1. Effect of PBAN on Pheromone Production

#### 3.1.1. Virgin Females

**Figure 1:**
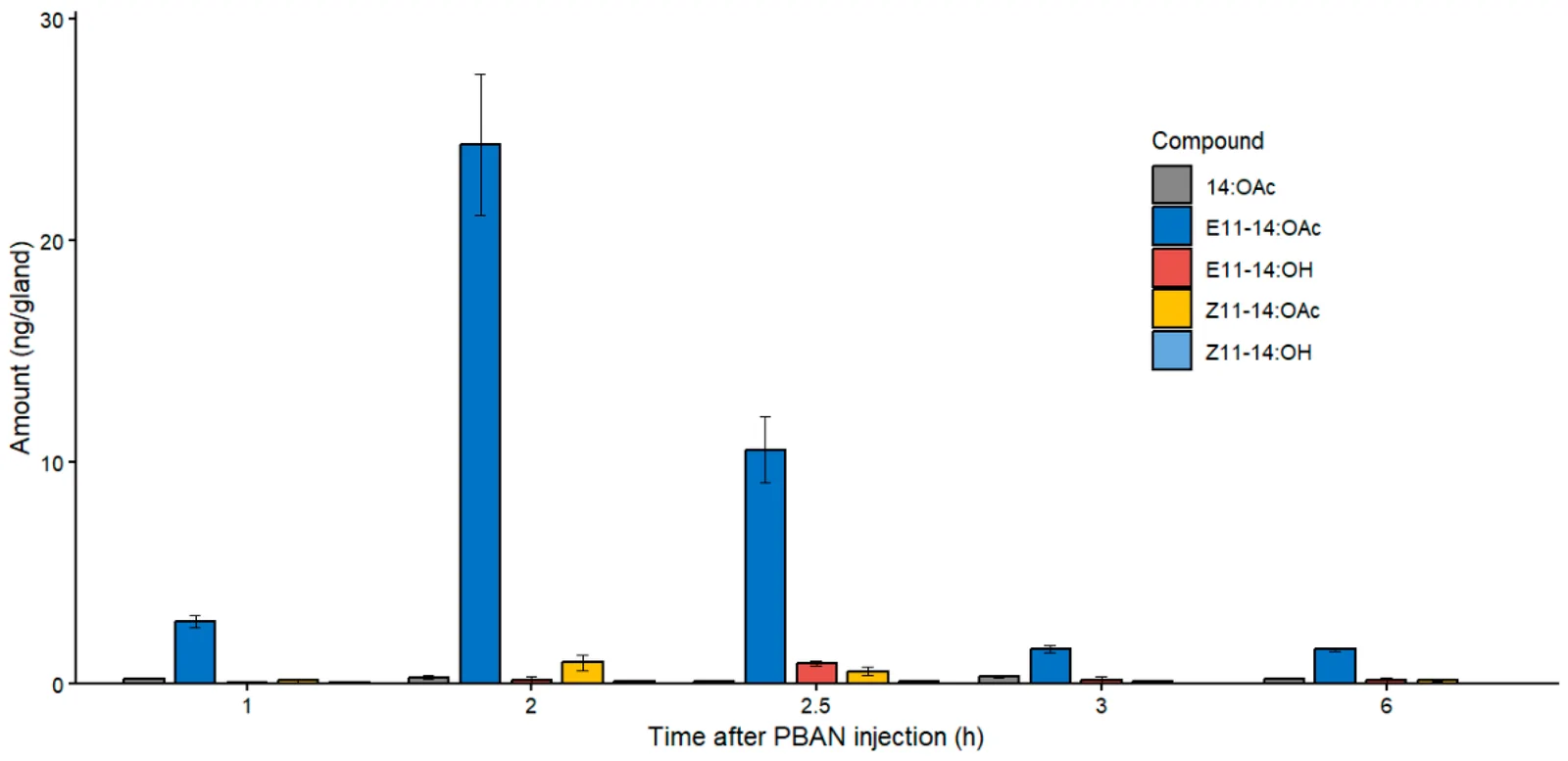
PBAN-induced production of pheromone compounds in virgin females of *P. auraria* (*n =* 3). Bars represent mean amounts (ng/gland) ± SD (error bars) for each compound.

PBAN application to virgin females of *P. auraria* induced a marked increase in the production of the major pheromone component, E11-14:OAc. At 1 h post-treatment, the amount was 2.83 ± 0.25 ng/gland, while at 2 h, the highest production was reached, with an average of 24.32 ± 2.94 ng/gland (maximum observed value of 26.31 ng/gland), which was a marked increase over baseline levels. At 2.5 h, the amount decreased to 10.57 ± 1.40 ng/gland and then declined to levels near the initial values, with mean values of 1.59 ± 0.16 ng/gland at 3 h and 1.57 ± 0.10 ng/gland at 6 h. The minor components (E11-14:OH, Z11-14:OAc, 14:OAc, and Z11-14:OH) were detected at much lower amounts (always ≤0.9 ng/gland) and showed less pronounced temporal variations (Figure 1). In control females (which were injected with PBS without PBAN during the photophase), only traces of all compounds (≤0.08 ng) were observed at all time points.

#### 3.1.2. Mated Females

**Figure 2:**
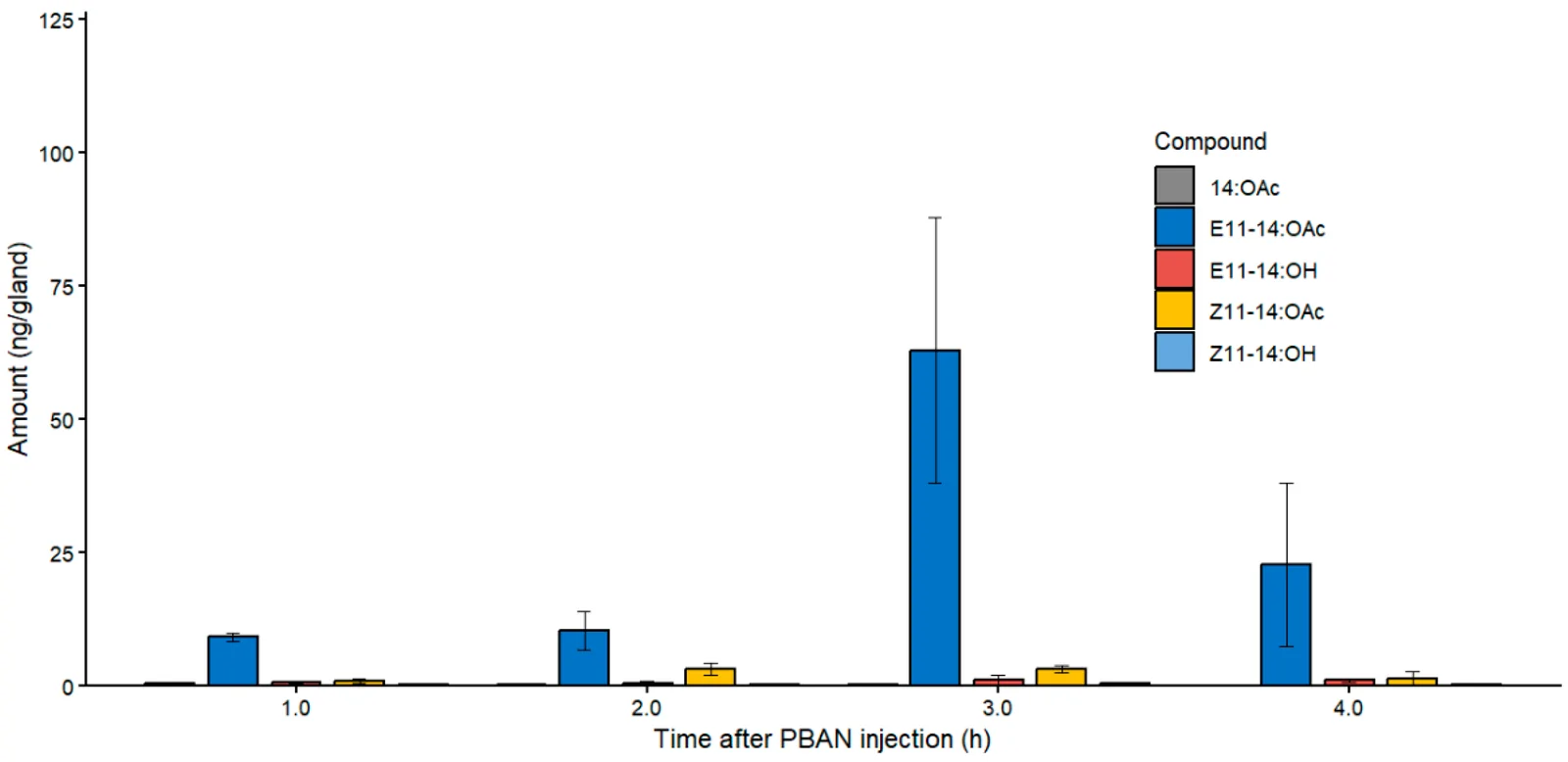
PBAN-induced production of pheromone compounds in mated females of *P. auraria*. Bars represent mean amounts (ng/gland) ± SD (error bars) for each compound based on analyses of three individual females per sampling time (*n =* 3).

PBAN application in mated females also stimulated pheromone production, although with different temporal patterns and high variability among replicates. The major component E11-14:OAc averaged 9.22 ± 0.75 ng/gland at 1 h and 10.41 ± 3.39 ng/gland at 2 h, followed by a highly variable peak of 62.95 ± 25.09 ng/gland at 3 h, which decreased to 22.72 ± 15.66 ng/gland at 4 h (Figure 2). The other components remained at low levels (≤4.4 ng/gland). Control females produced only traces (≤0.08 ng) of all compounds.

### 3.2. Fatty Acid Composition of the Pheromone Gland

The fatty acids of the pheromone gland of *P. auraria* were analyzed as their methyl esters after derivatization with BF_3_-methanol. Comparison of retention times on an RTX-5 column and mass spectra with those of authentic standards identified 23 compounds (Table 1). The most abundant methyl esters were (*Z*)-9-octadecenoate (oleate, ≈50%), hexadecanoate (palmitate, ≈30%), and (*Z*)-9-hexadecenoate (palmitoleate, ≈4%). In addition, methyl (*E*)-11-tetradecenoate was detected at a low but constant proportion (≈0.08%), while its (*Z*)-11-tetradecenoate isomer was observed only at trace levels (≈0.03%). Other saturated and unsaturated esters with chain lengths from C10 to C22 were also identified in variable amounts (Table 1).

### 3.3. Incorporation of Deuterium-Labeled Fatty Acids

**Figure 3:**
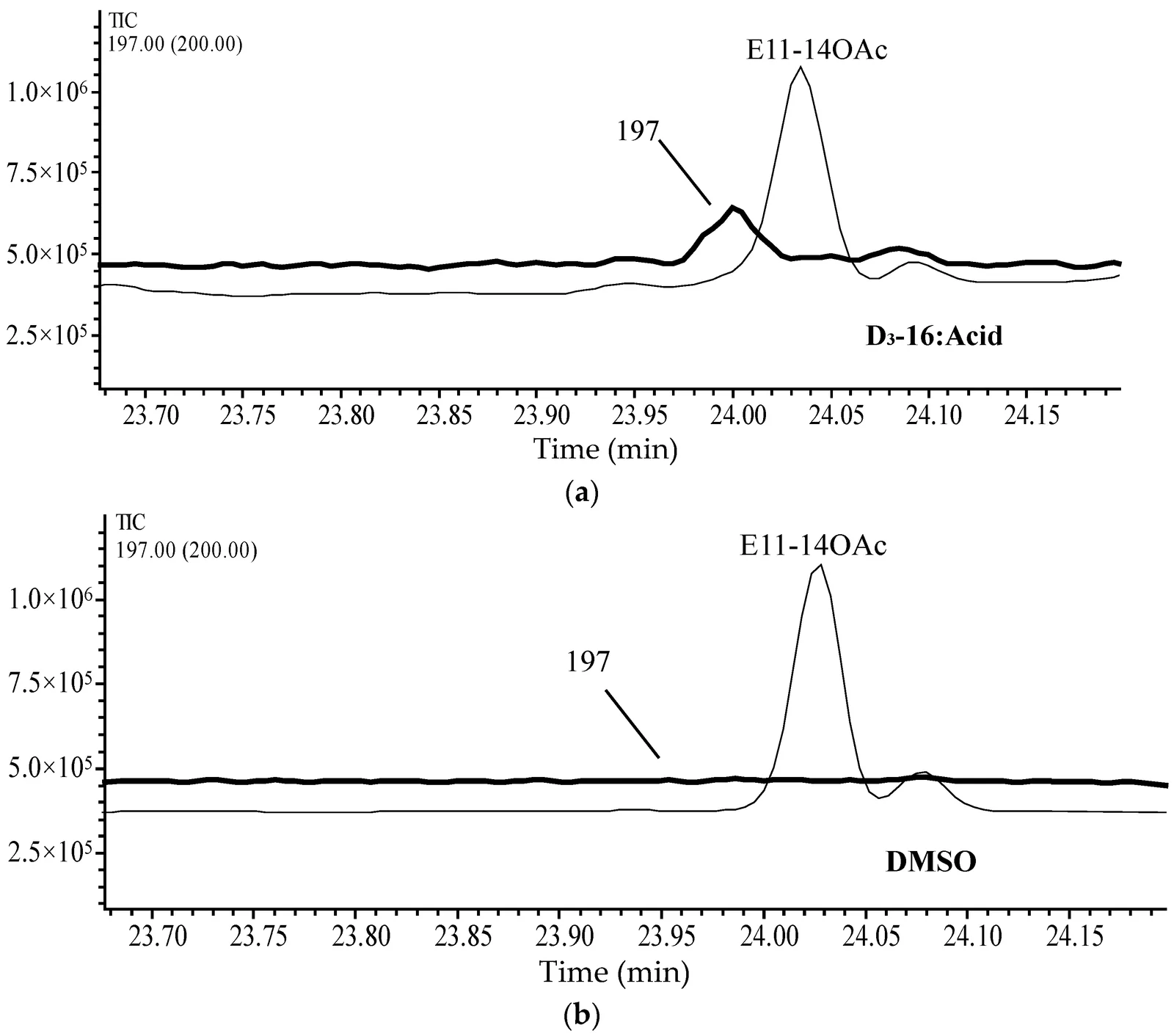
Incorporation of deuterium labeling from topically applied D_3_-16:acid into the pheromone component of *P. auraria*. (**a**) Female treated with D_3_-16:acid + PBAN, showing ions at *m*/*z* 194 and 197. (**b**) Control female (DMSO + PBAN), showing only the ion at *m*/*z* 194.

In the mass spectrum of the major pheromone component, (*E*)-11-tetradecenyl acetate (E11-14:OAc), an additional ion at *m*/*z* 197 was observed alongside the characteristic ion at *m*/*z* 194 of the natural compound (Figure 3a). In control extracts (DMSO), only the ion at *m*/*z* 194 appeared (Figure 3b). This shift (194 + 3) is indicative of deuterium incorporation and was detected in 4 out of 6 females treated with D_3_-16:acid, 4 out of 6 treated with D_3_-14:acid, and 4 out of 7 treated with D_3_-18:acid. In contrast, no shifted ions were observed in the minor components (E11-14:OH, Z11-14:OAc, 14:OAc, and Z11-14:OH) in any of the treated females. The molecular ion of the deuterated acetate (expected M^+^ at *m*/*z* 257) was very weak and was not considered diagnostic.

**Figure 4:**
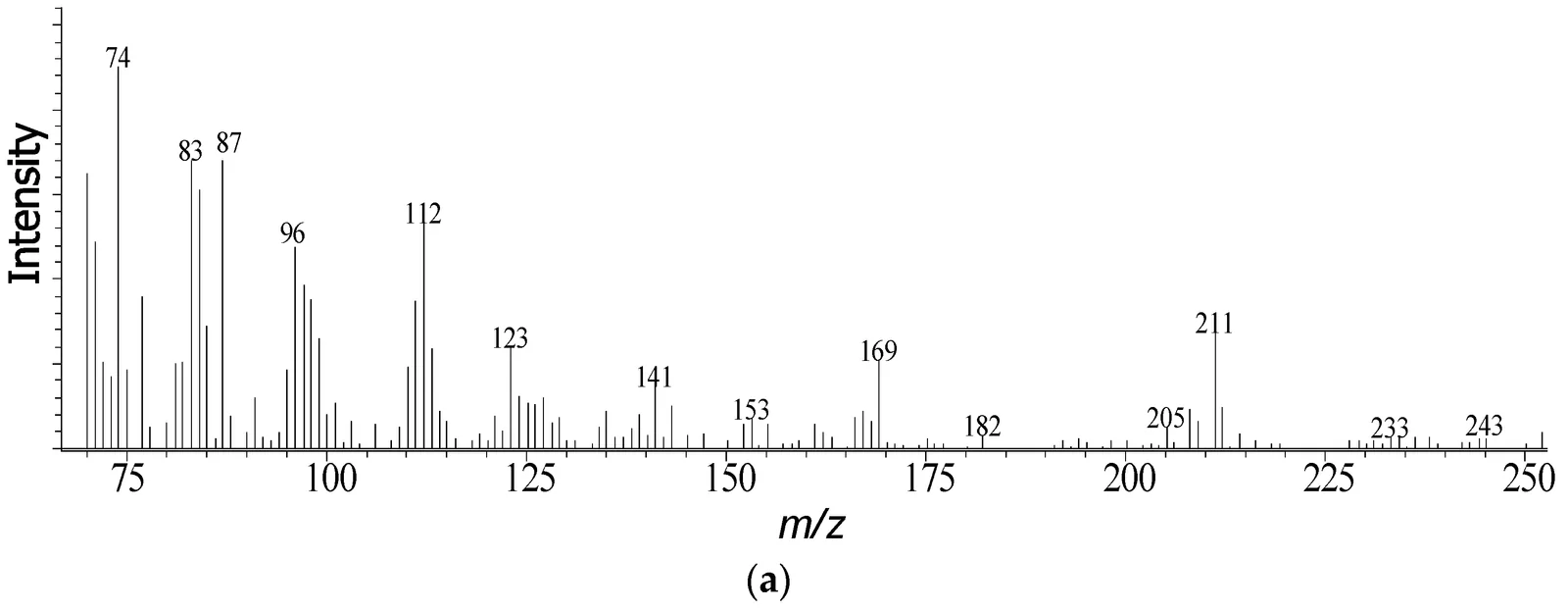

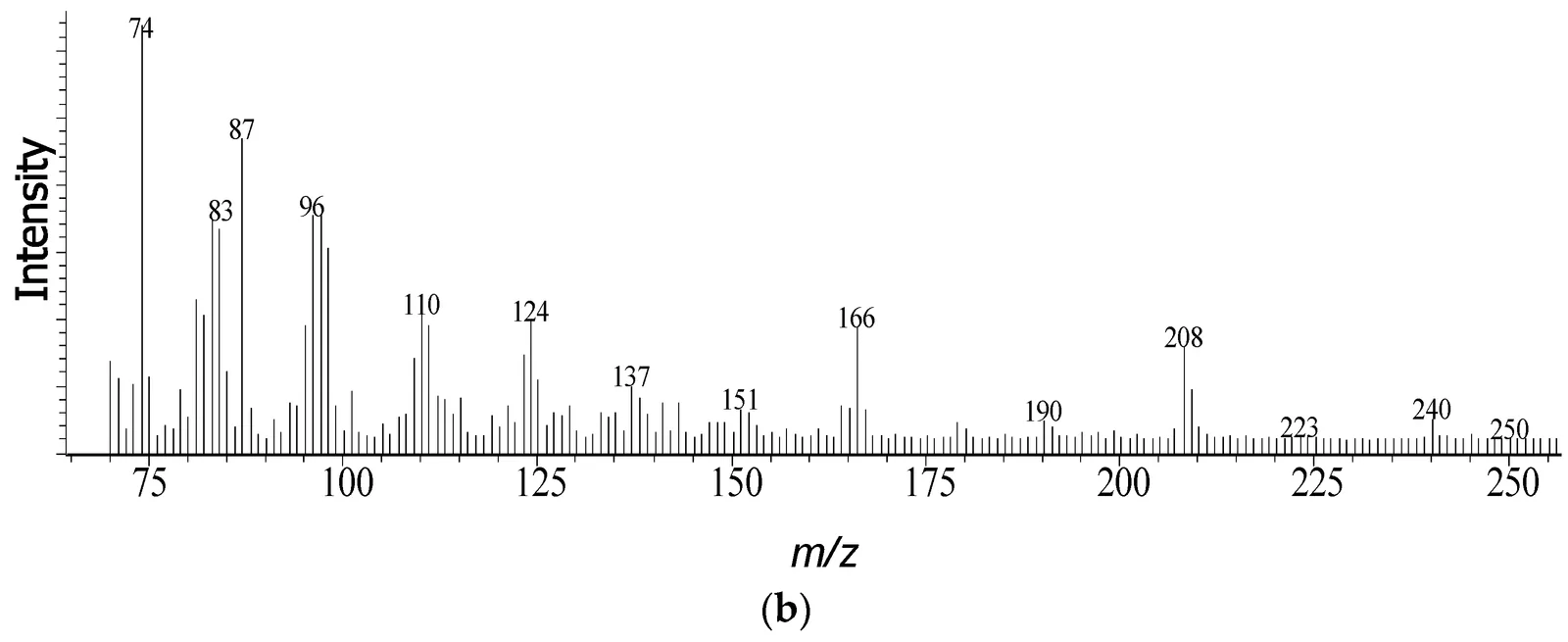
Mass spectra of methyl (E)-11-tetradecenoate (E11-14:COOMe) from *P. auraria*. (**a**) Female treated with D_3_-16:acid + PBAN. Deuterated ions at *m*/*z* 169 and 211 are observed. (**b**) Control (DMSO + PBAN), showing natural ions at *m*/*z* 166 and 208. The molecular ion (M^+^) is very weak due to the low amount of compound.

In methylated extracts with BF_3_-methanol, the detection of deuterated methyl (*E*)-11-tetradecenoate (E11-14:COOMe) was less frequent. The shifted ions at *m*/*z* 169 and 211 (corresponding to the [M–74]^+^ and [M–32]^+^ fragments of the deuterated compound) were observed only in 2 out of 6 females treated with D_3_-16:acid and in 1 out of 7 treated with D_3_-18:acid. In females treated with D_3_-14:acid (0 out of 6) and in the remaining samples, only the natural ions at *m*/*z* 166 and 208 were detected. Figure 4a shows a representative spectrum from a female treated with D_3_-16:acid, where the deuterated ions at *m*/*z* 169 and 211 are clearly visible, whereas in the control (Figure 4b), only the natural ions at *m*/*z* 166 and 208 appear. The deuterated molecular ion at *m*/*z* 243 was visible but at low intensity, appearing only in the positive samples.

**Figure 5:**
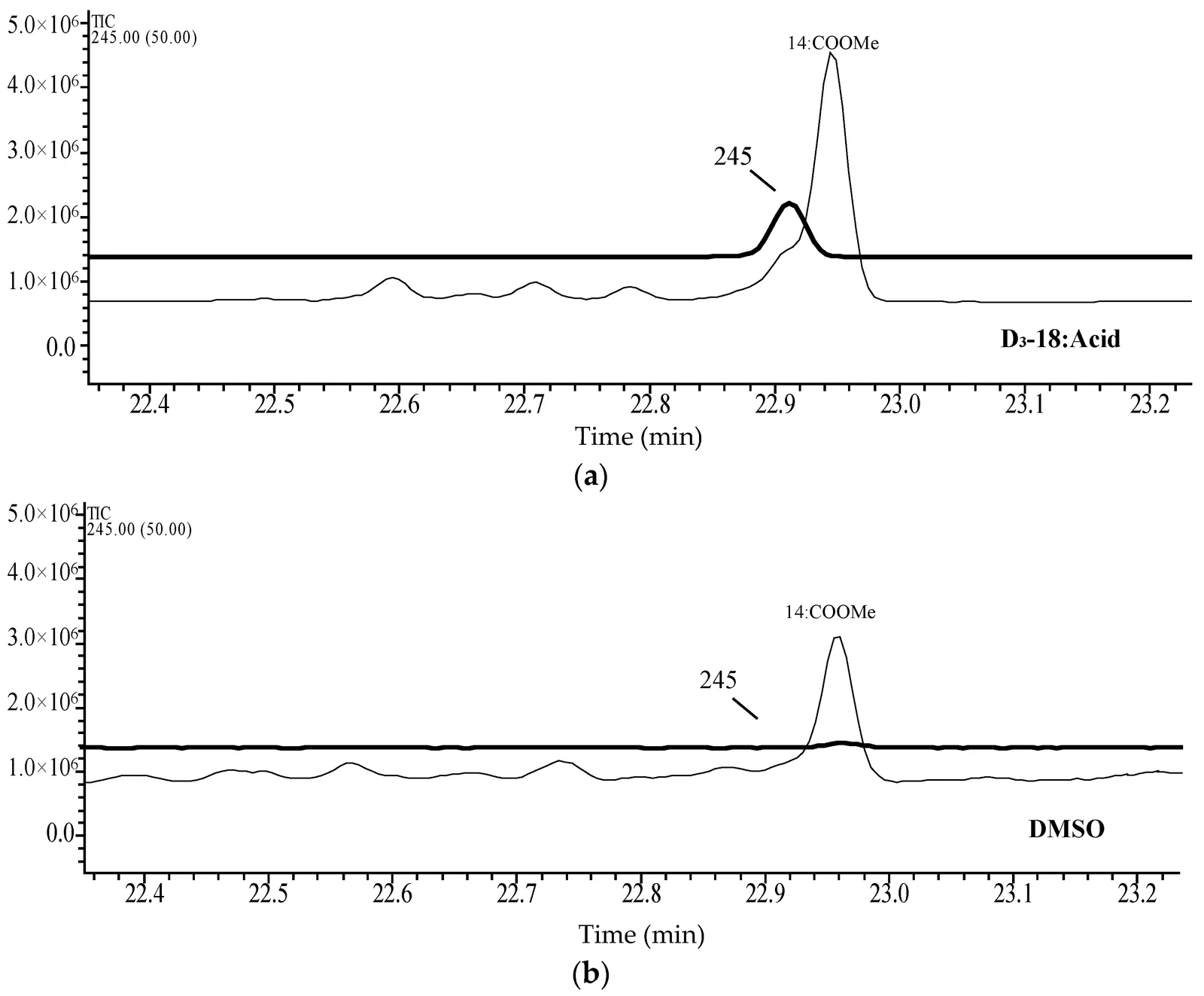
Full-scan chromatograms of methylated extracts from pheromone glands of *P. auraria*. (**a**) Female treated with D_3_-16:acid + PBAN: M^+^ at *m*/*z* 245 (D_3_-14:COOMe). (**b**) Control (DMSO + PBAN): peak with M^+^ at *m*/*z* 242.

In methylated extracts from females treated with D_3_-16:acid, an additional peak was detected that co-eluted with natural methyl tetradecanoate (14:COOMe) (with a slightly shorter retention time) in 4 out of 6 treated females. Its molecular ion appeared at *m*/*z* 245 (Figure 5). This peak was identified as the deuterated analogue D_3_-14:COOMe and was not observed in control extracts. Furthermore, similar incorporation was obtained in 5 out of 7 females treated with D_3_-18:acid.

## 4. Discussion

### 4.1. PBAN Selectively Regulates the Major Pheromone Component

Although the sample size used in this study (*n* = 3) precludes inferential statistical analysis and limits the estimation of variability, the descriptive results reveal a large biological response. Nevertheless, the magnitude of the observed effect is consistent with the robust action of PBAN in other Lepidoptera [5,6]. In virgin females of *P. auraria*, PBAN injection during the photophase induced a marked increase in the major component E11-14:OAc, averaging 24.32 ± 2.94 ng/gland at 2 h post-injection (Figure 1). This value exceeds the maximum natural amount reported for untreated virgin females during calling behavior in the scotophase (19.43 ± 0.54 ng/gland) [21]. Control females injected with PBS during the photophase showed only trace amounts (≤0.08 ng), confirming that PBAN is required to stimulate pheromone production outside the natural calling period under the experimental conditions tested. In contrast, the minor components (E11-14:OH, Z11-14:OAc, 14:OAc, and Z11-14:OH) remained at very low levels (always ≤0.9 ng/gland), well below the described natural values. Although the cold anesthesia protocol [22] could constitute a minor limitation, previous observations in the same species showed no effect of cold immobilization on pheromone production compared to non-anesthetized females [21]. Both control and PBAN-treated females were subjected to the same immobilization procedure, supporting comparative analysis.

Despite the PBAN-induced increase in pheromone titers, typical calling behavior was not observed in virgin females during the photophase, indicating a decoupling between pheromone biosynthesis and behavioral emission, even in unmated females. It has been documented in the scientific literature that although PBAN is the main regulator of pheromone biosynthesis in lepidopterans, its mode of action is not homogeneous for all components of the blend. For example, in *Dendrolimus punctatus* (Lepidoptera, Lasiocampidae), PBAN injection induces the production of a compound (Z5,E7-12:Ald) that is not detected in normal females [26]. These findings indicate that the pheromone profile induced by PBAN can differ qualitatively from the natural one and that the regulation of minor components may depend on other factors that are not directly activated by the neuropeptide [5,6].

PBAN injection in mated females caused a highly variable increase in pheromone production, with some females reaching exceptionally high amounts of up to 90 ng per gland (Figure 2). Although these values suggest mating may alter PBAN sensitivity, the sample size precludes statistical comparison. This high variability and delayed response profile explain why different sampling schedules were used; virgin females (1–6 h) exhibited a sharp peak followed by a rapid return to baseline, whereas mated females (1–4 h) displayed a delayed and highly variable response, necessitating different time-course windows. Females were designated as “mated” based on direct observation of copulatory behavior, although dissection was not performed to confirm the presence of a spermatophore in the bursa copulatrix to optimize the use of specimens. Therefore, observed copulation does not necessarily confirm successful insemination. Despite their variability, these values are comparable to those reported in other tortricids, where pheromone content in the gland usually ranges from 1 ng to 220 ng per female [27,28]. As in virgin females, the minor components were not affected by PBAN injection in mated females (Figure 2). Similarly, no calling behavior was observed in mated females after PBAN injection. This decoupling between pheromone biosynthesis and behavioral emission is consistent with reports in other tortricids, where PBAN has been shown to act directly on the pheromone gland and stimulate synthesis, while calling has an independent neural control mechanism [29].

### 4.2. Fatty Acid Profile of the Pheromone Gland

Analysis of methyl esters from the pheromone gland of *P. auraria* revealed a profile where palmitate (C16:0), oleate (Z9-18:1), and palmitoleate (Z9-16:1) were predominant, together with smaller amounts of stearate (C18:0) and linoleate (Z9,Z12-18:2). This pattern is consistent with a previous report [14] and is typical of tortricids with Type I pheromones, where long-chain saturated fatty acids act as biosynthetic precursors [4,5].

The presence of methyl (*E*)-11-tetradecenoate (E11-14:COOMe) in a low proportion (≈0.08%) and traces of its Z isomer (≈0.03%) provide relevant evidence for the biosynthetic pathway. This compound is proposed as the immediate acyl precursor of the pheromone E11-14:OAc. The strong dominance of the E isomer over the Z in the methyl ester (≈2.7:1) coincides with the pheromone composition (100:4) and suggests a highly stereospecific Δ11 desaturase, similar to those described in other tortricids [30,31]. The small amount of Z11-14:COOMe could be due to secondary activity of the desaturase or to an additional low-expression desaturase.

The final E/Z ratio is mainly determined by the selectivity of the desaturase, although an additional filter at the reductase or acetyltransferase level cannot be ruled out [32]. Moreover, the presence of methyl tetradecanoate (14:COOMe, ≈0.5%) indicates that myristic acid is available as a substrate. Labeling experiments with D_3_-16:acid confirmed that palmitic acid is shortened to 14:CoA, demonstrating that β-oxidation contributes to the generation of the substrate for desaturation, a common pathway in tortricids [4,10]. The longer-chain (C20, C22) and polyunsaturated esters are not considered relevant for pheromone biosynthesis.

### 4.3. Isotopic Labeling and Biosynthetic Pathway

**Figure 6:**
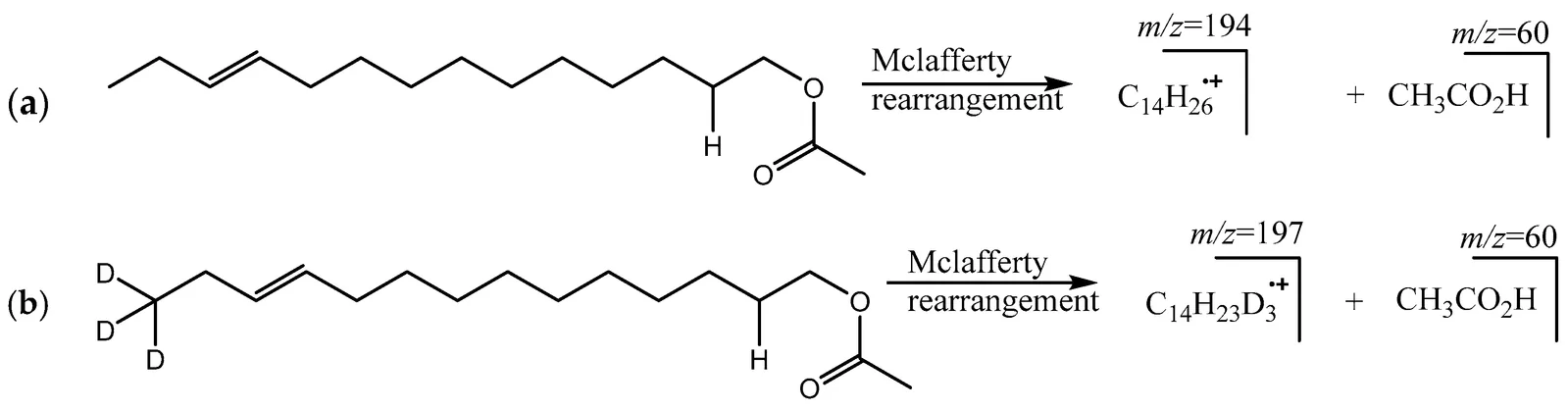
McLafferty rearrangement of (*E*)-11-tetradecenyl acetate (E11-14:OAc) and its deuterated analogue. (**a**) Natural compound: the rearrangement produces a fragment ion at *m*/*z* 194, corresponding to the loss of acetic acid (CH_3_CO_2_H). (**b**) D_3_-labeled compound (deuterium atoms at the terminal methyl group): the same rearrangement produces a fragment ion shifted to *m*/*z* 197, consistent with the retention of the three deuterium atoms in the hydrocarbon fragment.

In the mass spectra of E11-14:OAc obtained from females treated with D_3_-16:acid, an additional ion at *m*/*z* 197 was observed alongside the characteristic ion at *m*/*z* 194 of the natural compound (Figure 3). The ion at *m*/*z* 197 corresponds to the fragment generated by McLafferty rearrangement, which shifted relative to the natural ion (194 + 3). This indicates that the three deuteriums of the precursor’s terminal methyl group were retained in the hydrocarbon chain during fragmentation (Figure 6). In control extracts (females injected only with DMSO), only the ion at *m*/*z* 194 was detected, with no signal at *m*/*z* 197.

In a previous study, methyl (*E*)-11-tetradecenoate was identified as a gland component, whereas the corresponding Z isomer was not detected, suggesting a strong stereochemical bias in the biosynthetic pathway [21]. In the present work, traces of the (*Z*)-11-tetradecenoate isomer were detected (Table 1), indicating that the Δ11 desaturase of *P. auraria* produces a small proportion of the Z isomer. This is consistent with the pheromone composition, where Z11-14:OAc is approximately 4% of the major component.

GC-MS analysis of methylated extracts from glands treated with D_3_-16:acid revealed diagnostic ions indicating the incorporation of the deuterated precursor into monounsaturated tetradecenoate intermediates. In the case of methyl (*E*)-11-tetradecenoate, ions were detected at *m*/*z* 211 and 169 and correspond to shifts of +3 Da relative to the natural ions at *m*/*z* 208 and 166, respectively. These labeled ions were detected at the expected retention times and were absent in control extracts, which confirms that deuterium from D_3_-16:acid is incorporated into the Δ11 unsaturated intermediates involved in pheromone biosynthesis.

Additionally, in methylated extracts from females treated with D_3_-16:acid, peak co-elution with natural methyl tetradecanoate (14:COOMe) was detected in 4 out of 6 treated females, with its molecular ion appearing at *m*/*z* 245 (Figure 5), which identified it as the deuterated analogue D_3_-14:COOMe. This peak was absent in control extracts. This observation demonstrates that deuterated palmitic acid is shortened to deuterated tetradecanoic acid without loss of labeling at the terminal methyl group. The same incorporation was confirmed in females treated with D_3_-18:acid.

**Figure 7:**
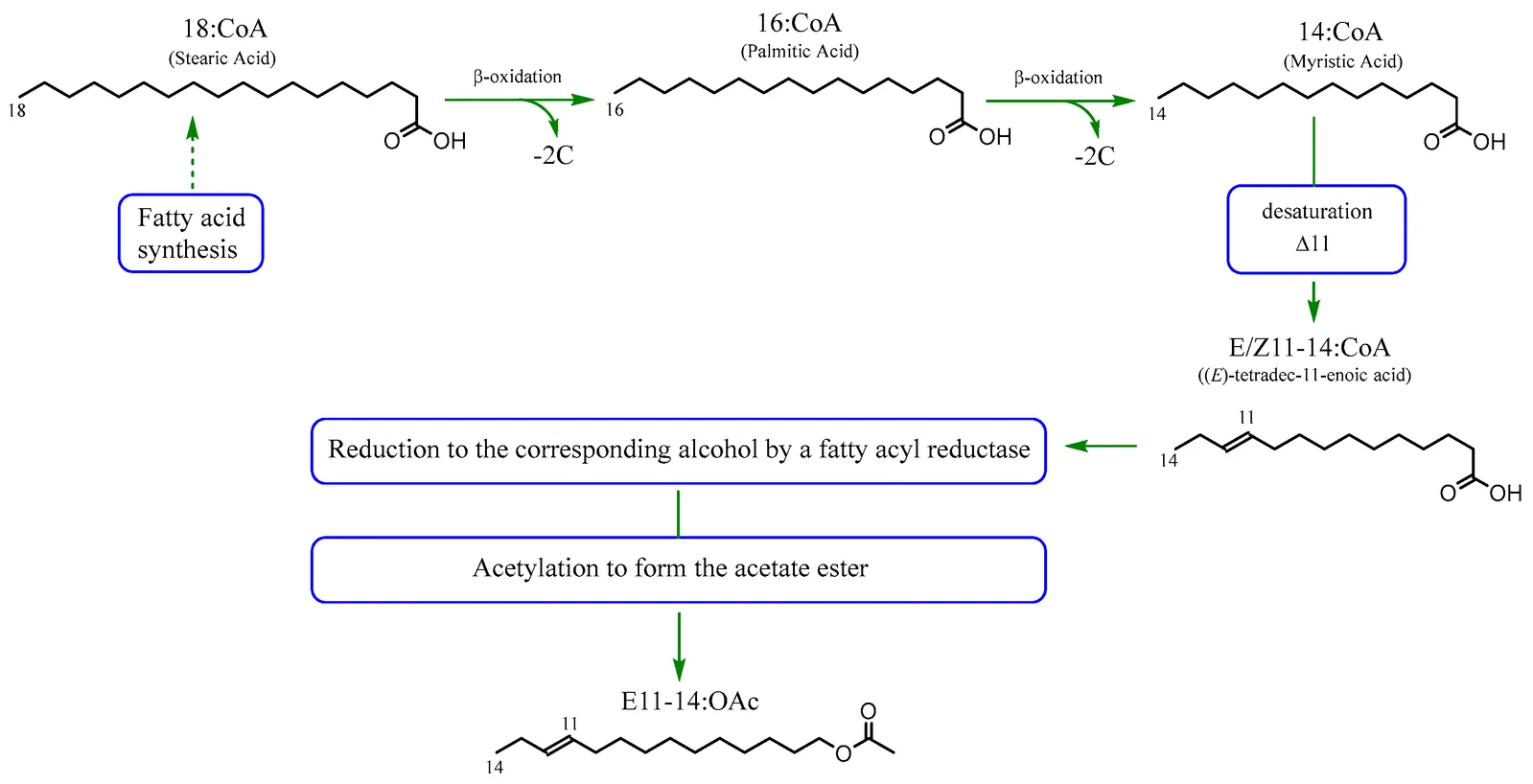
Proposed biosynthetic pathway for the major sex pheromone component (*E*)-11-tetradecenyl acetate (E11-14:OAc) in *P. auraria*. The pathway involves sequential β-oxidation of stearic acid, Δ11 desaturation, reduction to the corresponding alcohol, and acetylation.

Together, these results are consistent with a pathway in which palmitic and stearic acids are shortened by β-oxidation from the carboxyl end, followed by a highly stereoselective Δ11 desaturation toward the *E* isomer, and finally reduction and acetylation to produce E11-14:OAc. This mechanism combining chain shortening by β-oxidation with Δ11 desaturation has been documented in other tortricids [2,4,32,33,34,35]. Based on these findings, the biosynthetic pathway outlined in Figure 7 is proposed.

## 5. Conclusions

This study provides the first direct evidence of the biosynthetic origin of the major pheromone component of *P. auraria*, (*E*)-11-tetradecenyl acetate. PBAN injection markedly increased pheromone production in virgin females, whereas mated females exhibited high variability. In both virgin and mated females, the increase in pheromone titers did not translate into calling behavior, indicating decoupling between biosynthesis and behavioral emission that appears independent of mating status. The fatty acid profile of the pheromone gland revealed the presence of the presumed acyl precursor (*E*)-11-tetradecenoate and only traces of its Z isomer, suggesting a highly stereospecific Δ11 desaturase.

Labeling experiments with [16,16,16-^2^H_3_]palmitic acid provided evidence that chain shortening from C16 to C14 occurs through β-oxidation from the carboxyl end, as evidenced by the retention of three deuteriums in the terminal methyl group of E11-14:OAc (shift in the ion from *m*/*z* 194 to 197) and the detection of D_3_-14:COOMe (M^+^ at *m*/*z* 245). Similar incorporation was observed with D_3_-18:stearic acid. These findings allow us to propose a biosynthetic pathway involving β-oxidation, Δ11 desaturation, reduction, and acetylation. This work contributes to the knowledge of pheromone communication in Tortricidae and could provide a basis for the biotechnological production of this pheromone for pest management.

## Figures and Tables

**Table 1:** Fatty acid methyl esters identified in methylated extracts of the pheromone gland of *P. auraria*.

No.	Retention Time	Compound	Relative Amount (%) ^a^
1	15.11	Methyl nonanoate	0.01 ± 0.01
2	16.92	Methyl decanoate	0.01 ± 0.01
3	20.18	Methyl dodecanoate	0.17 ± 0.02
4	23.02	Methyl (*E*)-11-tetradecenoate	0.08 ± 0.01
5	23.04	Methyl (*Z*)-11-tetradecenoate	0.03 ± 0.01
6	23.08	Methyl tetradecanoate	0.51 ± 0.03
7	24.04	Methyl (*Z*)-5-tetradecenoate	0.02 ± 0.01
8	24.08	Methyl (*Z*)-9-tetradecenoate	0.01 ± 0.01
9	24.42	Methyl pentadecanoate	0.24 ± 0.02
10	25.38	Methyl (*E*)-9-hexadecenoate	0.01 ± 0.01
11	25.40	Methyl (*Z*)-9-hexadecenoate	0.02 ± 0.01
12	25.44	Methyl (*E*)-11-hexadecenoate	0.36 ± 0.04
13	25.47	Methyl (*Z*)-11-hexadecenoate	4.14 ± 0.21
14	25.78	Methyl hexadecanoate	29.92 ± 2.39
15	26.66	Methyl 10-heptadecenoate	0.33 ± 0.03
16	26.92	Methyl heptadecanoate	0.44 ± 0.04
17	27.49	Methyl (9*Z*,12*Z*,15*Z*)-9,12,15-octadecatrienoate	0.01 ± 0.01
18	27.82	Methyl (9*Z*,12*Z*)-9,12-octadecadienoate	0.08 ± 0.01
19	27.82	Methyl (*E*)-9-octadecenoate	8.61 ± 0.69
20	27.93	Methyl (*Z*)-9-octadecenoate	49.97 ± 4.00
21	28.11	Methyl octadecanoate	4.25 ± 0.21
22	30.27	Methyl eicosanoate	0.38 ± 0.04
23	32.29	Methyl docosanoate	0.41 ± 0.04

^a^ Values are mean ± SD of eight individual pheromone glands. For mass spectra see Supplementary Material Figure S1.

## Data Availability

The original contributions presented in this study are included in the article. Further inquiries can be directed to the corresponding author.

## Supplementary Materials

The following supporting information can be downloaded at: https://www.mdpi.com/article/10.3390/insects17101037/s1. Figure S1: Full-scan chromatogram of fatty acid methyl esters (FAMEs) from the pheromone gland of *Proeulia auraria*; Figure S2: Mass spectra of each FAME identified in Table 1.

## Abbreviations

The following abbreviations are used in this manuscript:

DMSO	Dimethyl sulfoxide
DMDS	Dimethyl disulfide
E11-14:OAc	(*E*)-11-Tetradecenyl acetate
E11-14:OH	(*E*)-11-Tetradecenol
FID	Flame ionization detector
GC	Gas chromatography
GC-MS	Gas chromatography–mass spectrometry
M^+^	Molecular ion
PBAN	Pheromone-biosynthesis-activating neuropeptide
PBS	Phosphate-buffered saline
SD	Standard deviation
SAG	Chilean Agricultural and Livestock Service (Servicio Agrícola y Ganadero)
Z11-14:OAc	(*Z*)-11-Tetradecenyl acetate
Z11-14:OH	(*Z*)-11-Tetradecenol
14:OAc	Tetradecyl acetate
14:COOMe	Methyl tetradecanoate
D_3_-14:acid	[14,14,14-^2^H_3_]Tetradecanoic acid
D_3_-16:acid	[16,16,16-^2^H_3_]Hexadecanoic acid (palmitic acid)
D_3_-18:acid	[18,18,18-^2^H_3_]Octadecanoic acid (stearic acid)
