# Supplementary material for: Effect of PBAN on Pheromone Production and Biosynthetic Pathway of the Major Sex Pheromone Component of Proeulia auraria Clarke (Lepidoptera: Tortricidae)
Source: Insects. 2026 Oct 9;17(10):1037. doi: 10.3390/insects17101037 (PMC13705223; doi:10.3390/insects17101037)

Figure S1. Representative GC–MS chromatogram of fatty acid methyl esters (FAMEs) identified in methylated extracts of the pheromone gland of P. auraria.

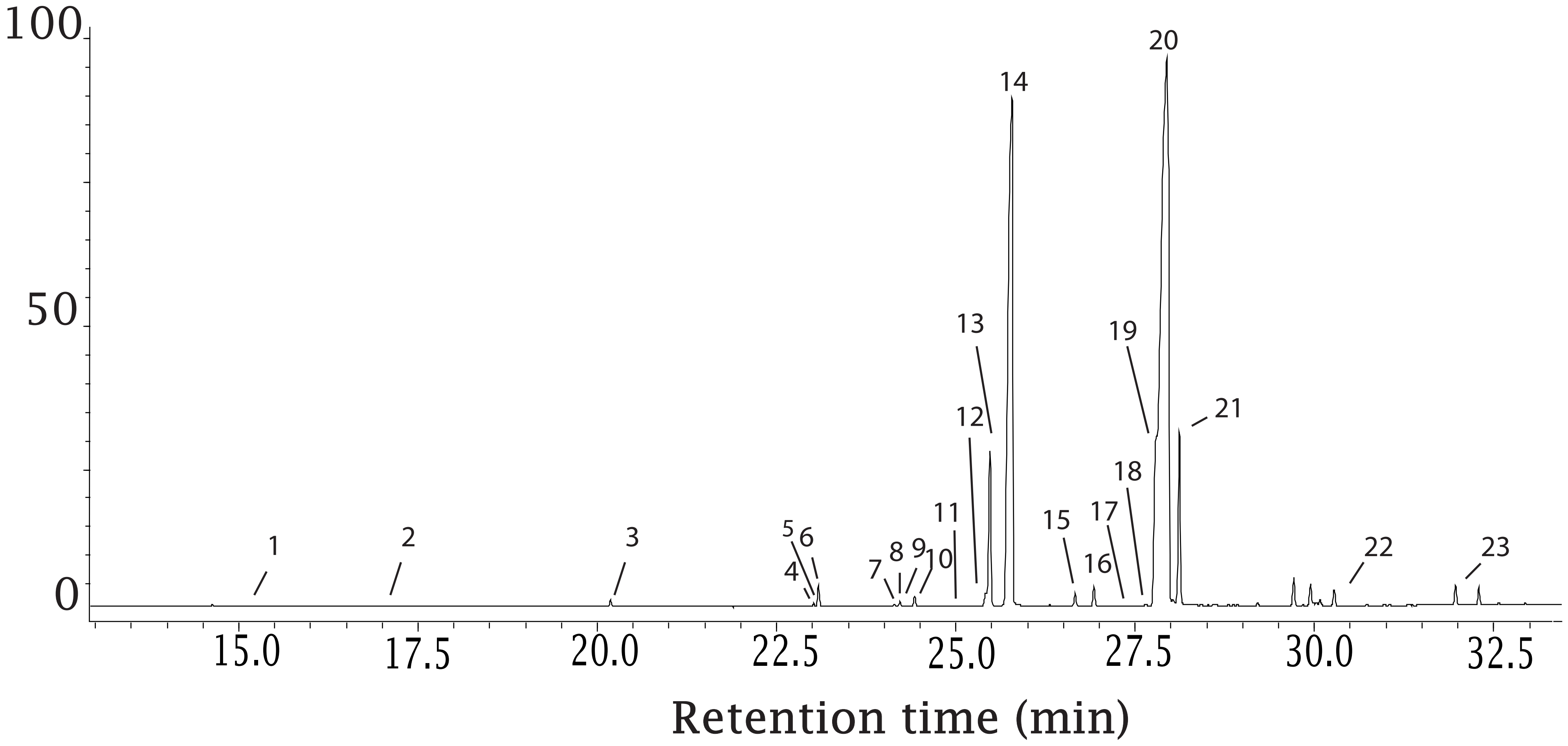

Supplement: Supplementary file 1 [file insects-17-01037-s001.zip › Figure S1 Full-scan chromatogram of fatty acid methyl esters FAMEs from the phero-mone gland of Proeulia auraria.pdf]
