# Supplementary material for: Effect of PBAN on Pheromone Production and Biosynthetic Pathway of the Major Sex Pheromone Component of Proeulia auraria Clarke (Lepidoptera: Tortricidae)
Source: Insects. 2026 Oct 9;17(10):1037. doi: 10.3390/insects17101037 (PMC13705223; doi:10.3390/insects17101037)
Supplement: Supplementary file 1 [file insects-17-01037-s001.zip › Figure S2 Mass spectra of each FAME identified in Table 1.pdf]

Figure S2. Electron impact mass spectra (70 eV) of fatty acid methyl esters (FAMEs) identified in methylated extracts of the pheromone gland of Proeulia auraria.

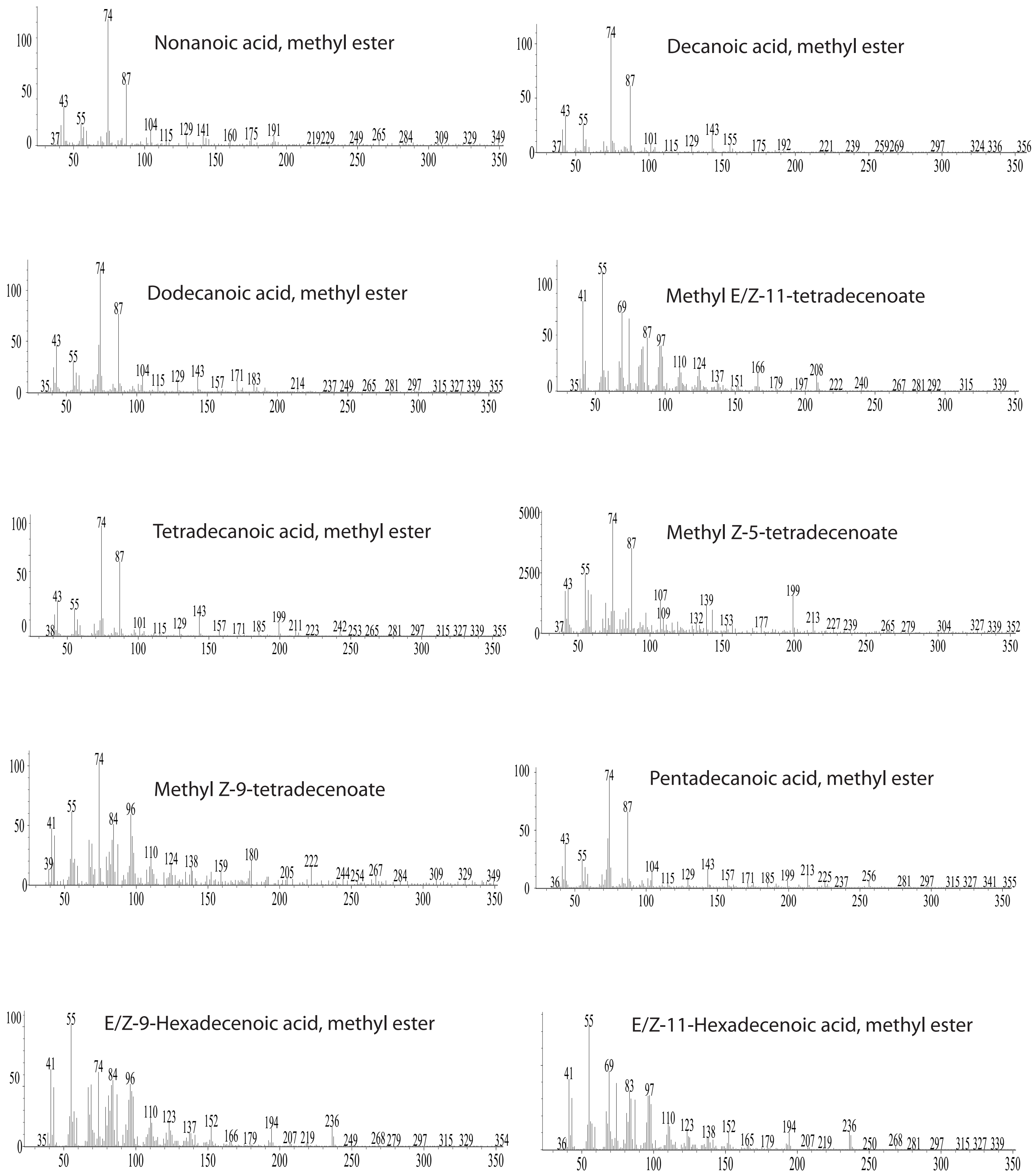

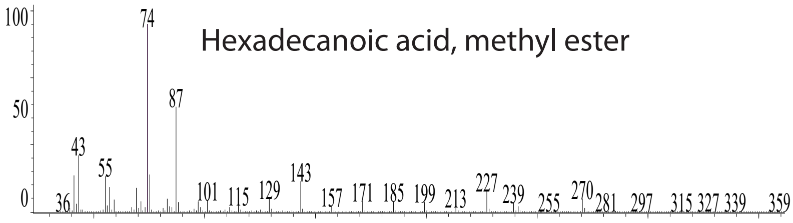
Hexadecanoic acid, methyl ester

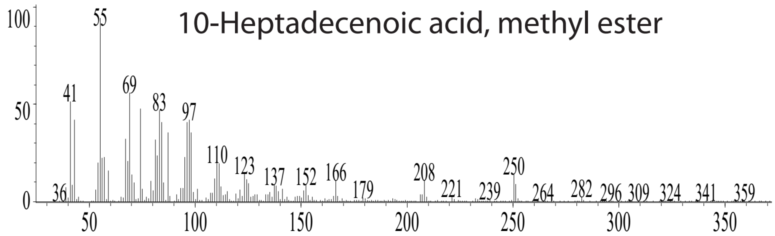
10-Heptadecenoic acid, methyl ester

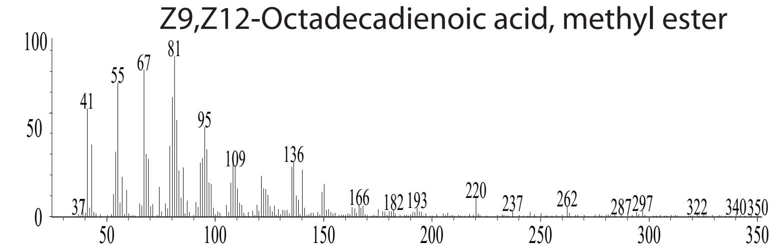
Z9,Z12-Octadecadienoic acid, methyl ester

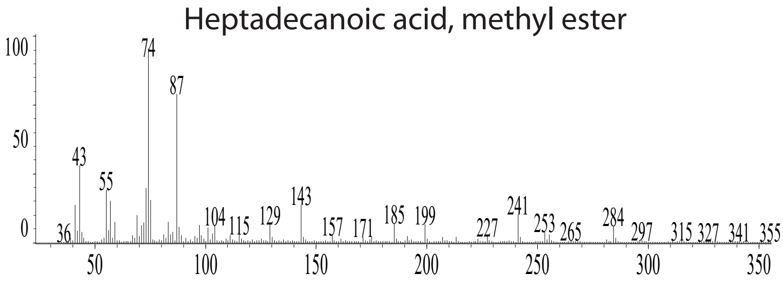
Heptadecanoic acid, methyl ester

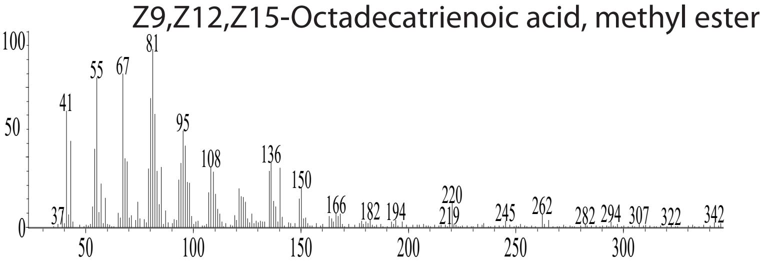
Z9,Z12,Z15-Octadecatrienoic acid, methyl ester

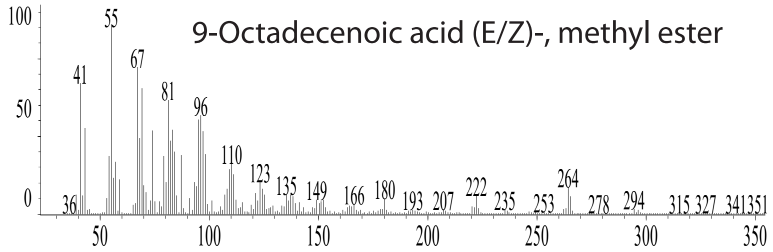
9-Octadecenoic acid (E/Z)-, methyl ester

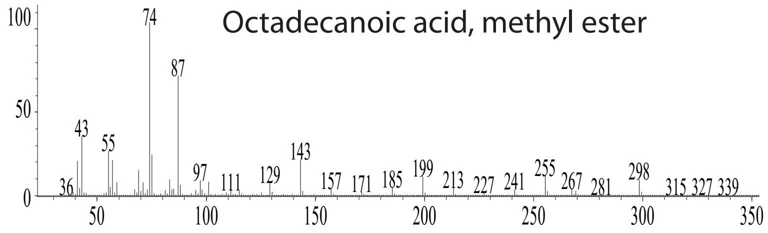
Octadecanoic acid, methyl ester

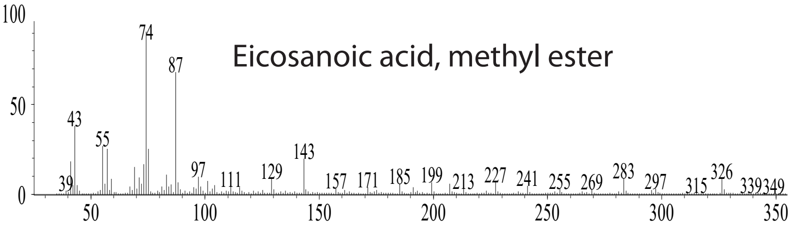
Eicosanoic acid, methyl ester

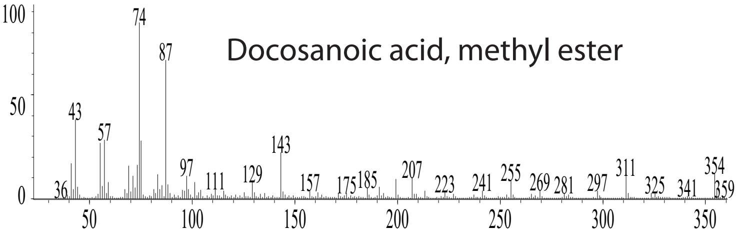
Docosanoic acid, methyl ester
